# Negative impact of asthma on patients in different age groups*

**DOI:** 10.1590/S1806-37132015000100003

**Published:** 2015

**Authors:** Marcela Batan Alith, Mariana Rodrigues Gazzotti, Federico Montealegre, James Fish, Oliver Augusto Nascimento, José Roberto Jardim

**Affiliations:** University of São Paulo, University Hospital, São Paulo, Brazil. Pulmonary Rehabilitation Center, Federal University of São Paulo Paulista School of Medicine; and Physical Therapist. University of São Paulo University Hospital, São Paulo, Brazil; São Camilo University Center, São Paulo, Brazil. Pulmonary Rehabilitation Center, Federal University of São Paulo Paulista School of Medicine; and Professor of Physical Therapy. São Camilo University Center, São Paulo, Brazil; University of Puerto Rico, School of Public Health, Reio Piedras, PR, USA. Merck, Sharp & Dohme Corp., Carolina, PR, USA; and Professor. University of Puerto Rico School of Public Health, Reio Piedras, PR, USA; Sharp & Dohme Corp., Whitehouse Station, NJ, USA, Global Scientific Affairs. Merck, Sharp & Dohme Corp., Whitehouse Station (NJ) USA; Federal University of São Paulo, Paulista School of Medicine, São Paulo, Brazil. Federal University of São Paulo Paulista School of Medicine, São Paulo, Brazil; Federal University of São Paulo, Paulista School of Medicine, São Paulo, Brazil, Federal University of São Paulo Paulista School of Medicine, São Paulo, Brazil

**Keywords:** Asthma, Age groups, Quality of life

## Abstract

**Objective:**

To evaluate the impact of asthma on patients in Brazil, by age group (12-17 years, 18-40 years, and ≥ 41 years).

**Methods:**

From a survey conducted in Latin America in 2011, we obtained data on 400 patients diagnosed with asthma and residing in one of four Brazilian state capitals (São Paulo, Rio de Janeiro, Curitiba, and Salvador). The data had been collected using a standardized questionnaire in face-to-face interviews. For the patients who were minors, the parents/guardians had completed the questionnaire. The questions addressed asthma control, number of hospitalizations, number of emergency room visits, and school/work absenteeism, as well as the impact of asthma on the quality of life, sleep, and leisure. We stratified the data by the selected age groups.

**Results:**

The proportions of patients who responded in the affirmative to the following questions were significantly higher in the 12- to 17-year age group than in the other two groups: "Have you had at least one episode of severe asthma that prevented you from playing/exercising in the last 12 months?" (p = 0.012); "Have you been absent from school/work in the last 12 months?" (p < 0.001); "Have you discontinued your asthma relief or control medication in the last 12 months?" (p = 0.008). In addition, 30.2% of the patients in the 12- to 17-year age group reported that normal physical exertion was very limiting (p = 0.010 vs. the other groups), whereas 14% of the patients in the ≥ 41-year age group described social activities as very limiting (p = 0.011 vs. the other groups).

**Conclusions:**

In this sample, asthma had a greater impact on the patients between 12 and 17 years of age, which might be attributable to poor treatment compliance.

## Abstract (pt)

**Objetivo:**

Avaliar o impacto da asma em pacientes segundo as faixas etárias de 12-17 anos, 18-40 anos e ≥ 41 anos no Brasil.

**Métodos:**

Os dados de 400 pacientes com asma diagnosticada por um médico e residentes de quatro capitais estaduais brasileiras (São Paulo, Rio de Janeiro, Curitiba e Salvador) foram obtidos em um inquérito realizado em países da América Latina em 2011. Os dados foram coletados por meio de um questionário padronizado em entrevista presencial com os pacientes ou com os pais/responsáveis daqueles < 18 anos. As questões abordavam controle da asma, número de hospitalizações, número de consultas de urgência, absenteísmo na escola/trabalho e impactos da asma na qualidade de vida, sono e lazer. Os dados foram estratificados pelas faixas etárias selecionadas.

**Resultados:**

Em comparação com os grupos de pacientes adultos, houve uma proporção significativamente maior no grupo 12-17 anos em relação a ter ao menos um episódio de asma grave que impediu o paciente a continuar a jogar ou a se exercitar nos últimos 12 meses (p = 0,012), absenteísmo escolar/trabalho nos últimos 12 meses (p < 0,001), e interrupção de medicação para controle ou prevenção da asma nos últimos 12 meses (p = 0,008). Além disso, 30,2% dos pacientes na faixa etária 12-17 anos relataram que esforços físicos normais eram atividades muito limitantes (p = 0,010 vs. outros grupos), enquanto 14% dos pacientes do grupo ≥ 41 anos descreveram as atividades sociais como muito limitantes (p = 0,011 vs. outros grupos).

**Conclusões:**

Nessa amostra, o impacto da asma foi maior nos pacientes com idade entre 12 e 17 anos do que nos adultos, e isso pode ser atribuído à baixa aderência ao tratamento.

## Introduction

According to the World Health Organization, an estimated 235 million people worldwide have asthma.^(^ 1 ^)^ In Brazil, the prevalence of clinically diagnosed asthma is approximately 20%, and the frequency of active disease is 10%, rates that are not very different from those found in developed countries.^(^ 2 ^)^ Asthma has a major impact on public and private health care systems in Brazil. 

Asthma is one of the leading chronic diseases of childhood and is considered the most prevalent chronic respiratory disease in children and adolescents. It is a potentially serious condition, the prevalence of which has increased worldwide.^(^ 3 ^)^ It affects not only children but also adults, being a global health problem. Fortunately, hospitalization rates for asthma in individuals over 20 years of age decreased by 49% between 2000 and 2010. In 2011, the Brazilian Unified Health Care System Department of Information Technology recorded 160,000 hospitalizations among patients of all ages, asthma being therefore the fourth leading cause of hospitalizations.^(^ 4 ^)^ Factors associated with an increased risk of symptom persistence into adulthood include disease severity, atopy, smoking, and being female.^(^ 5 ^)^

Because asthma is a chronic disease, patients must adhere to medication and guidelines for the self-management of asthma, which include general information on asthma, written action plans for asthma, and asthma symptom diaries. All of the above are very effective in reducing morbidity and mortality in asthma patients.^(^ 6 ^)^

The Global Initiative for Asthma classifies asthma patients as having controlled, partly controlled, or uncontrolled asthma on the basis of symptoms, limitations in activities of daily living, nocturnal awakenings, rescue medication use, and pulmonary function data; in addition, it states the importance of achieving and maintaining clinical control of asthma as a treatment goal.^(^ 1 ^,^ 7 ^,^ 8 ^)^ To that end, it is necessary to know the extent to which each goal is met; to identify barriers to asthma control; and to determine whether subgroups of patients stratified by age are at an increased risk because of poor disease control.^(^ 1 ^)^

This knowledge would allow us to determine the impact of asthma on the quality of life of patients in different age groups and provide appropriate guidance to each group. Our hypothesis was that the level of asthma control was higher in patients between 12 and 17 years of age because they are cared for and supervised by their parents/caregivers. The objective of the present study was to evaluate the impact of asthma on the quality of life of 400 asthma patients residing in one of four Brazilian state capitals, interviewed in person, and stratified by age group (12-17 years, 18-40 years, and ≥ 41 years). 

## Methods

The Latin America Asthma Insight and Management (LA AIM) survey was conducted in 2011 in Argentina, Brazil, Mexico, Venezuela, and Puerto Rico in order to explore and document patient perception of asthma, as well as patient knowledge of the disease and its forms of treatment.^(^ 9 ^)^ The present study is a cross-sectional study using a subsample of the multicenter LA AIM survey and focusing exclusively on the patients residing in Brazil. The present study was approved by the Research Ethics Committee of the Federal University of São Paulo *Hospital São Paulo* (Ruling no. 250.155). 

Initially, 4,545 households in four Brazilian cities (São Paulo, Rio de Janeiro, Curitiba, and Salvador) were selected from a national probability sample. If there were two or more individuals with asthma in the household, one of them was randomly selected. All of the individuals who reported having physician-diagnosed asthma were included. A home visit was scheduled over the telephone, and individuals were interviewed by a professional interviewer trained to administer the questionnaire. A total of 400 individuals were interviewed in person; those who were ≥ 18 years of age were interviewed directly, as were the parents/guardians of those who were between 12 and 17 years of age. The interviews lasted approximately 35 minutes. The questionnaire consisted of 53 questions addressing five major asthma domains: symptoms; impact of asthma on life; perception of asthma control; exacerbations; and treatment/medication.^(^ 10 ^)^

In order to evaluate the impact of asthma on the daily life of respondents, the questions addressed the frequency of school or work absences due to asthma, activity limitations because of the disease, productivity levels on days when experiencing an asthma attack, and the influence of asthma on the quality of life. Respondents were also asked whether they or their children had been hospitalized or had been admitted to the ICU in the last 12 months and, if so, how many times. In addition, respondents were asked whether they had sought a physician for exacerbations, symptoms of worsening disease, and severe asthma attacks in the previous year. Respondents also answered questions regarding their (or their children's) treatment. 

In the statistical analysis, categorical variables are presented as absolute numbers and percentages, and continuous variables are presented as mean and standard deviation. The chi-square test was used in order to compare categorical variables among the age groups studied (12-17 years, 18-40 years, and ≥ 41 years), and the level of significance was set at p < 0.05. Data analysis was performed with the Statistical Package for the Social Sciences, version 18.0 (SPSS Inc., Chicago, IL, USA). 

## Results

We evaluated 400 asthma patients residing in one of four cities in Brazil: São Paulo (47.8%); Rio de Janeiro (36.0%); Curitiba (7.0%); and Salvador (9.2%). Of the 400 patients, 128 (32%) were male and 272 (68%) were female. Most of the patients ≥ 41 years of age were female (p = 0.011). Of the sample as a whole, approximately half had pets in the household (p = 0.037). There were no significant differences among the three age groups regarding the presence of smokers in the household or a history of rhinitis or allergy (Table 1). 

**Table 1 -:** Demographic and clinical characteristics of 400 asthma patients interviewed in one of four Brazilian state capitals and stratified by age group.a

Variable	Age group, years			p*
	12-17	18-40	≥ 41	
	(n = 43)	(n = 185)	(n = 172)	
Gender				
Female	20 (46.5)	126 (68.1)	121 (70.3)	0.011
Male	23 (53.5)	59 (31.9)	51 (29.7)	
Pets in the household	24 (55.8)	86 (46.5)	89 (52.0)	0.037
Smokers in the household	24 (55.8)	80 (43.2)	68 (39.5)	0.155
History of rhinitis or allergy	33 (76.7)	143 (77.3)	127 (73.8)	0.738
Level of asthma control^b^				
Controlled	3 (7.0)	24 (13.0)	10 (5.8)	0.197
Partly controlled	26 (60.5)	98 (53.0)	102 (59.3)	
Uncontrolled	14 (32.6)	63 (34.1)	60 (34.9)	

a Values expressed as n (%).

b In accordance with the Global Initiative for Asthma criteria.(7)

* Chi-square test.

Regarding activities of daily living, the proportion of patients who had had at least one episode of severe asthma that prevented them from playing/exercising in the last 12 months was significantly higher in the 12- to 17-year age group than in the other two groups. Of the asthma patients in the 12- to 17-year age group, 67.4% had been absent from school or work in the last 12 months (p < 0.001). There were no significant differences among the groups regarding the other activities of daily living (Table 2). 

**Table 2 -:** Activities of daily living affected by asthma in 400 asthma patients interviewed in one of four Brazilian state capitals and stratified by age group.a

Variable	Age group, years			p*
	12-17	18-40	≥ 41	
	(n = 43)	(n = 185)	(n = 172)	
Episode of severe asthma that prevented the patient from playing or exercising	23 (53.5)	76 (41.1)	52 (30.2)	0.012
Episode of severe asthma that forced the patient to leave school or work	24 (55.8)	93 (50.3)	63 (36.6)	0.104
School or work absenteeism	29 (67.4)	75 (40.5)	48 (27.9)	< 0.001

a Values expressed as n (%).

* Chi-square test.

Regarding activities limited by asthma (Table 3) in the 12- to 17-year age group (n = 43), 13 (30.2%) reported that normal physical exertion was limiting (p = 0.010), whereas 24 (14.0%) of the patients in the ≥ 41-year age group (n = 172) described social and daily activities as limiting (p = 0.011 and p = 0.005, respectively). 

**Table 3 -:** Activities of daily living limited by asthma in 400 asthma patients interviewed in one of four Brazilian state capitals and stratified by age group.a

Variable	Age group, years			p*
	12-17	18-40	≥ 41	
	(n = 43)	(n = 185)	(n = 172)	
Sports and recreation	12 (28.0)	24 (13.0)	34 (19.8)	0.197
Normal physical exertion	13 (30.2)	23 (12.5)	42 (24.5)	0.010
Social activities	5 (11.6)	10 (5.5)	24 (14.0)	0.011
Sleep	14 (32.6)	46 (24.9)	49 (28.5)	0.220
Daily activities	7 (16.3)	21 (11.4)	35 (20.3)	0.005

a Values expressed as n (%).

* Chi-square test.

There were no differences among the groups regarding episodes of asthma requiring hospitalization or emergency room visit (Table 4). 

**Table 4 -:** Episodes of asthma requiring hospital admission, ICU admission, unscheduled urgent visit, or emergency room visit in 400 asthma patients interviewed in one of four Brazilian state capitals and stratified by age group.a

Variable	Age group, years			p*
	12-17	18-40	≥ 41	
	(n = 43)	(n = 185)	(n = 172)	
Episode of severe asthma requiring hospital admission	28 (65.1)	115 (62.2)	85 (49.4)	0.071
Episode of severe asthma requiring ICU admission	2 (4.7)	11 (5.9)	13 (7.6)	0.914
Emergency visit to a physician’s office, a hospital, or a clinic	22 (51.2)	86 (46.5)	79 (45.9)	0.824
Emergency room visits or hospitalizations	13 (30.2)	56 (30.3)	40 (23.3)	0.297
Most commonly used health service				
Private	6 (14.0)	15 (8.1)	20 (11.6)	0.197
Health insurance	15 (34.8)	56 (30.3)	36 (20.9)	
PCC	11 (25.6)	77 (41.6)	83 (48.3)	
Other	11 (25.6)	37 (20.0)	33 (19.2)	

PCC: primary care clinic.

a Values expressed as n (%).

* Chi-square test.

Of the patients in the 12- to 17-year age group, 48.8% had discontinued their asthma relief or control medication in the last 12 months (p = 0.008; Table 5). 

**Table 5 -:** Questions regarding treatment in 400 asthma patients interviewed in one of four Brazilian state capitals and stratified by age group.a

Variable	Age group, years			p*
	12-17	18-40	≥ 41	
	(n = 43)	(n = 185)	(n = 172)	
Had heard of peak flow meters	22 (51.2)	64 (34.6)	66 (38.4)	0.334
Owned a peak flow meter	4 (9.3)	4 (2.1)	8 (4.6)	0.248
Had a written action plan for asthma management	19 (44.2)	75 (40.5)	74 (43.0)	0.210
Had used asthma relief or control medication in the last 4 weeks	12 (27.9)	42 (22.7)	50 (29.1)	0.496
Had discontinued asthma relief or control medication in the last 12 months	21 (48.8)	70 (37.8)	55 (31.9)	0.008

a Values expressed as n (%).

* Chi-square test.

## Discussion

The present study evaluated the impact of asthma on patients in three different age groups and showed that the disease had a greater impact on the patients between 12 and 17 years of age than on the adult patients. This finding was inconsistent with our hypothesis that the level of asthma control was higher in patients in the 12- to 17-year age group because they were cared for and supervised by their parents/caregivers. 

In our study, we found no significant differences among the age groups regarding the proportions of patients reporting the need for hospitalization, emergency room visits, or urgent physician visits for asthma; however, the proportions were high in the three age groups, ranging from 46.5% to 51.2% (Table 4). A multicenter prospective observational study involving a large cohort of asthma patients was conducted in the USA with the objective of gaining a better understanding of the natural history of asthma in patients with severe or difficult-to-treat asthma.^(^ 11 ^)^ The study found at least one hospitalization or emergency room visit in 5-15% of the adults, in 10-17% of the adolescents, and in 9-22% of the children,^(^ 11 ^)^ a finding that is inconsistent with those of the present study. This shows that the level of asthma control is low among asthma patients in Brazil.^(10)^

Of the asthma patients in the 12- to 17-year age group in the present study, 67.4% had been absent from school or work in the last 12 months (p < 0.001). Another survey of asthma patients in Latin America^(^ 12 ^)^ sought to assess the quality of asthma treatment and control in Latin America; to determine how closely asthma management guidelines were being followed; and to assess patient perception of, knowledge of, and attitudes toward asthma. In that survey, 79% of the adults and 68% of the children reported that asthma symptoms limited their activities of daily living; in addition, 58% of the children and 31% of the adults reported being absent from school or work because of asthma.^(^ 12 ^)^

A study conducted in California, USA, showed that school-age children (4-17 years of age) with daily or weekly asthma symptoms had a higher rate of missing at least one week of school in the last 12 months because of asthma (28%) than did children who experienced asthma symptoms less than once a month (15%). In adults with asthma, the rate of missing at least one week of work in the previous year because of asthma was more than twice as high among those with daily or weekly symptoms (12%) than among those who experienced symptoms less than once a month (5%).^(13)^

Work absenteeism reduces productivity and, consequently, increases the indirect costs of asthma. A study conducted in France and Spain with the objective of describing costs and quality of life in adult patients with asthma according to the level of asthma control found that patients with uncontrolled asthma increase asthma-related costs in the two countries.^(^ 8 ^)^

Given that asthma is a chronic disease, long-term treatment adherence is expected. Drug therapy is more effective when patients use corticosteroids regularly. However, many patients refuse to undergo long-term treatment. In addition, it is important to educate patients regarding the use of action plans and PEF diaries. This helps to reduce morbidity and mortality.^(^ 14 ^-^ 16 ^)^

In our study, there were no differences among the groups regarding the use of asthma relief or control medication (p = 0.496). However, 48.8% of the participants between 12 and 17 years of age had discontinued their asthma relief or control medication in the last 12 months (p = 0.008). There were no differences among the three groups of patients regarding their knowledge of the peak flow meter: approximately half of the patients knew what a peak flow meter was, but only less than 5% had one. In addition, 40% of the patients had received a written treatment plan, although, ideally, all should have. In a study conducted in India with the objective of investigating self-management in asthma patients, it was found that not all patients had metered dose inhalers at home, and only 2% had a peak flow meter and kept a PEF diary. In addition, none of the patients had received a written treatment plan from their physicians.^(6)^

In a study conducted in Michigan, USA, with the objective of evaluating adherence to treatment with inhaled corticosteroids in adult asthma patients (in the 18-50 year age bracket), adherence to treatment was found to be poor, being associated with a worse prognosis.^(^ 17 ^)^

It is known that adherence to inhaled corticosteroids is inadequate in Brazil and other countries.^(^ 18 ^-^ 20 ^)^ In a study conducted in the city of Belo Horizonte, Brazil, with the objective of evaluating the association between adherence to beclomethasone and the level of asthma control in children between 3 and 12 years of age, adherence to treatment was found to be very low, being a cause for concern.^(^ 21 ^)^ This finding is inconsistent with our hypothesis that the level of asthma control was higher in patients between 12 and 17 years of age because they are presumably cared for and supervised by their parents/caregivers. 

Our study has some limitations. Although we included patients residing in one four major cities in Brazil, it is possible that the study population does not represent the population with asthma in Brazil. However, it is unlikely that studies with this type of design can cover the entire population of a country. In addition, the data used in the present study were obtained from self-reports rather than medical records. Another limitation is the fact that parents/guardians completed the questionnaires for the participants between 12 and 17 years of age, which could have introduced an information bias. 

In the present study, we found that asthma had a greater impact on the patients between 12 and 17 years of age than on the adult patients. Therefore, we believe that young patients require appropriate counseling for a better understanding of their disease and the importance of adherence to treatment for asthma control. We also believe that programs for parents/caregivers should be developed so that these individuals can counsel their children on the importance of medication use. The opportunity to psychotherapy should also be provided to young patients in order to improve their treatment adherence and quality of life. 

In conclusion, asthma has a greater impact on young patients (between 12 and 17 years of age) than on adults, which might be attributable to poor treatment adherence. This shows that a more specific approach is required in order to improve treatment adherence among young patients. The negative impact of asthma on such patients includes lower participation in sports, physical activity, social activities, and daily activities, as well as school/work absenteeism. It is of note that, in the present study, the youngest group had the most contact with domestic animals, which can be a cause of poorer asthma control. 
